# Characterising Vascular Cell Monolayers Using Electrochemical Impedance Spectroscopy and a Novel Electroanalytical Plot [Retraction]

**DOI:** 10.2147/NSA.S326515

**Published:** 2021-06-29

Bussooa A. *Nanotechnol Sci Appl*. 2020;13:89–101.

The Editor-in-chief and Publisher of *Nanotechnology, Science and Applications* wish to retract the published paper. We were notified by the University of Glasgow’s Research Integrity Council that an investigation had found the scientific integrity of the paper had been compromised and it needed to be retracted. The investigation found the author had published data belonging to a group collaboration effort without proper authorisation, which included the use of the image shown in Figure 2. The author had published the paper under a grant he was not entitled to access and by publishing certain details within the paper the author had breached the University of Glasgow’s Intellectual Property polices.

The Editor has agreed with the request to retract the paper.

Our decision-making was informed by our policy on publishing ethics and integrity and the COPE guidelines on retraction.

The retracted article will remain online to maintain the scholarly record, but it will be digitally watermarked on each page as “Retracted”.

